# Supplementary material for: Predictive modeling of response to repetitive transcranial magnetic stimulation in treatment-resistant depression
Source: Transl Psychiatry. 2025 Apr 27;15:160. doi: 10.1038/s41398-025-03380-w (PMC12034820; doi:10.1038/s41398-025-03380-w)
Supplement: Supplementary file 1 — Supplemental Materials [file 41398_2025_3380_MOESM1_ESM.docx]

**Supplementary Materials**

**Coding and Categorization of Psychopharmacological Treatment Variables:**

Psychopharmacological treatment variables were recorded as binary indicators of concurrent use (coded as 1 for prescribed, 0 for not prescribed), and a daily dose burden was calculated for each category based on prescribed daily defined doses (DDD). Drug categories included antidepressants, benzodiazepines, non-benzodiazepine sedatives, anxiolytics, antipsychotics, lithium, non-lithium mood stabilizers, stimulants, opioids, and other psychopharmacological medications.

**
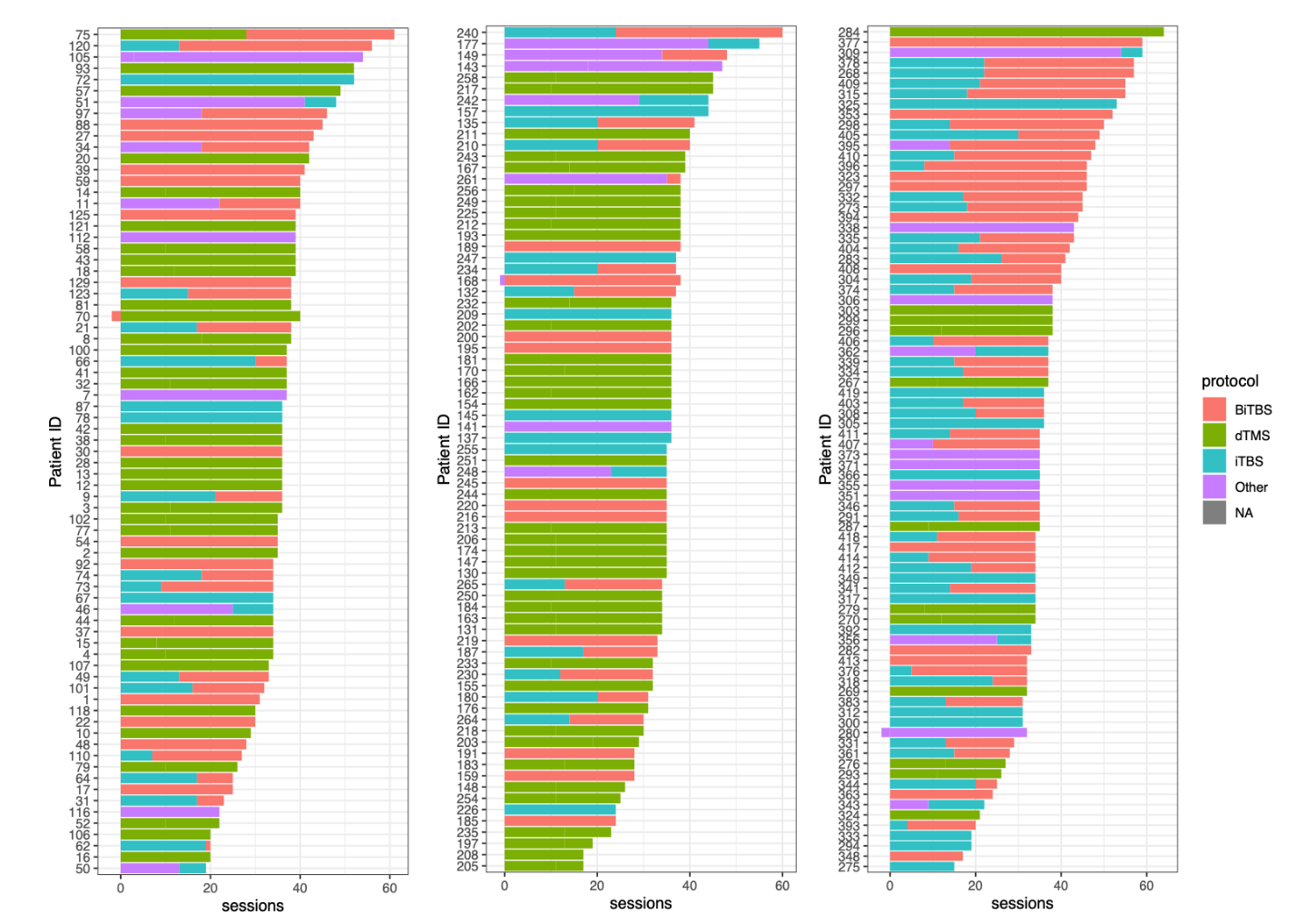
**

***Supplemental Figure 1.*** *rTMS protocol sequencing for all 232 patients included in the analytical sample. Within-patient changes in protocol are indicated by changes in color, and changes in the number of trains delivered per pulse within the same rTMS protocol are indicated by a broken line within the same color.*

**

***Supplemental Figure 2.*** *Permutation testing (B=99) of selected SVM classifier for treatment response (left, a) and the selected GBM classifier for depression remission (right, b). Red vertical line indicates generalization performance for classification (AUC) models with unpermuted data. Statistical significance is determined by the proportion of permuted samples (p) with superior AUC values relative to the true model (i.e., p / 100).*

***Supplemental Figure 3.*** *Mean generalization performance and 95% confidence intervals across (left panel) classification models in discriminating rTMS treatment remitters from non-remitters and (right panel) classification models in discriminating responders from non-responders. Results based on 20-(outer)fold nested cross-validation. Models using feature selection with univariate filters and ReliefF-based algorithms are shown in blue and red, respectively. SVM: support vector machine. RF: random forest. RFR: random forest regression. ENet: elastic net. GBM: gradient boosted machines. AUC: receiver operating characteristic area under the curve.*

***Supplemental Figure 4.*** *Calibration plots for the SVM classifier for treatment response (left, a) and the GBM classifier for depression remission (right, b). Predicted probabilities were calibrated using beta regression and binned into deciles (x-axis), then compared against observed probabilities within each bin (y-axis). Hosmer-Lemeshow (HL) test statistic shown to assess goodness-of-fit, as well as root mean squared error (RMSE). P-value derived from HL test.*

***
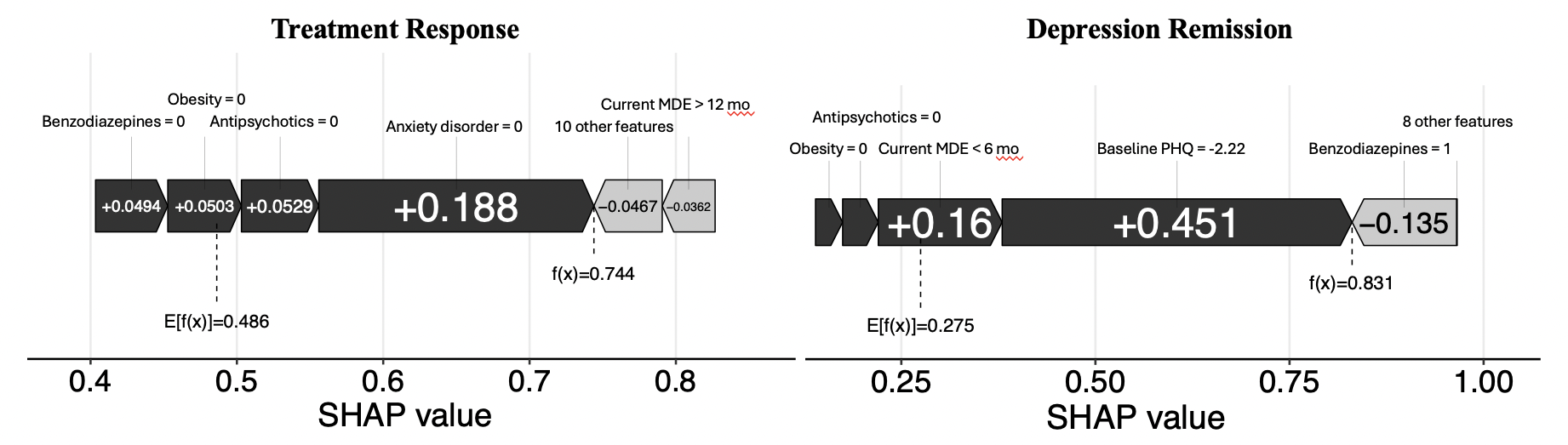
***

***Supplemental Figure 5.*** *SHAP force plots illustrating the additive contribution of individual features to the expected treatment response (a) or depression remission (b) probability for a representative patient from the analytical sample. Features shown in dark grey contribute positively to the patient’s response/remission probability, whereas those shown in light grey contribute negatively. (a) The sample mean expected treatment response probability, E[f(x)], is approximately 0.486 (or 48.6%), whereas for the depicted patient, without benzodiazepines, obesity, antipsychotic medication, or a comorbid anxiety disorder, but with a current major depressive episode (MDE) > 12 mo., their expected response probability, f(x), is 0.744 (or 74.4%). (b) The sample mean expected depression remission probability, E[f(x)], is approximately 0.275 (or 27.5%), whereas for the depicted patient, without obesity, antipsychotic medication, a current MDE < 6 mo., and a baseline PHQ-9 score that is 2.22 SD below the sample mean, but currently taking benzodiazepines, their expected remission probability, f(x), is 0.831 (or 83.1%).*

***Supplemental Figure 6.*** *Precision-recall (PR) curves for the response and remission models, with color gradients representing thresholds ranging from 0.0 (dark purple) to 1.0 (yellow). The PR AUC values are 0.51 and 0.40 for the response and remission models, respectively, indicating the models' precision-recall trade-offs given the imbalanced class distributions.*
